# Cisplatin attenuates taste cell homeostasis and induces inflammatory activation in the circumvallate papilla

**DOI:** 10.7150/thno.81153

**Published:** 2023-05-11

**Authors:** Wenwen Ren, Xudong Cha, Rui Xu, Tianyu Wang, Caiquan Liang, Janice Chou, Xiujuan Zhang, Fengzhen Li, Shenglei Wang, Boyu Cai, Peihua Jiang, Hong Wang, Huanhai Liu, Yiqun Yu

**Affiliations:** 1 Department of Otolaryngology, the Second Affiliated Hospital of the Naval Military Medical University (Shanghai Changzheng Hospital), Shanghai, People's Republic of China.; 2 Ear, Nose & Throat Institute, Department of Otolaryngology, Eye, Ear, Nose & Throat Hospital, Fudan University, Shanghai 200031, People's Republic of China.; 3 Olfactory Disorder Diagnosis and Treatment Center, Eye, Ear, Nose & Throat Hospital, Fudan University, Shanghai 200031, People's Republic of China; 4 School of Life Sciences, Shanghai University, Shanghai 200444, People's Republic of China.; 5 Monell Chemical Senses Center, Philadelphia, PA 19104, USA.

**Keywords:** Cisplatin, circumvallate papilla, taste receptor cell, inflammation, taste organoid, LY411575

## Abstract

**Rationale:** Gustation is important to several biological functions in mammals. However, chemotherapy drugs often harm taste perception in cancer patients, while the underlying mechanism is still unclear for most drugs and there is no effective way to restore taste function. This study investigated the effects of cisplatin on the taste cell homeostasis and gustatory function.

**Methods:** We used both mice and taste organoid models to study the effect of cisplatin on taste buds. Gustometer assay, gustatory nerve recording, RNA-Sequencing, quantitative PCR, and immunohistochemistry was performed to analyze the cisplatin-induced alteration in taste behavior and function, transcriptome, apoptosis, cell proliferation and taste cell generation.

**Results:** Cisplatin inhibited proliferation and promoted apoptosis in the circumvallate papilla, leading to significant impairment in taste function and receptor cell generation. The transcriptional profile of genes associated with cell cycle, metabolic process and inflammatory response was significantly altered after cisplatin treatment. Cisplatin inhibited growth, promoted apoptosis, and deferred taste receptor cell differentiation in taste organoids. LY411575, a γ-secretase inhibitor, reduced the number of apoptotic cells and increased the number of proliferative cells and taste receptor cells, potentially suggesting as a taste tissue protective agent against chemotherapy. LY411575 treatment could offset the increased number of Pax1^+^ or Pycr1^+^ cells induced by cisplatin in the circumvallate papilla and taste organoids.

**Conclusion:** This study highlights the inhibitory effects of cisplatin on taste cell homeostasis and function, identifies critical genes and biological processes regulated by chemotherapy, and proposes potential therapeutic targets and strategy for taste dysfunction in cancer patients.

## Introduction

Gustation is a crucial sense for both human and other animals, playing a vital role in nutrient intake, food digestion, and avoidance of potentially harmful or toxic foods 1. Taste disorders have a severe impact on human health and quality of life, resulting in anorexia, malnutrition, weight loss and even life-threatening conditions 2. While taste disorders such as ageusia, dysgeusia, and hypogeusia are relatively rare in the general population, they are prevalent among cancer patients, particularly those who receive radiation therapy or chemotherapy treatment for head and neck cancer 3. Approximately 50~80% of cancer patients experience taste alterations after chemotherapy treatment 4. Generally, cancer treatment blunts the sensitivity of taste buds for each tastant, including sweet, sour, and bitter. Moreover, abnormal taste sensation can serve as an early warning signal of tumor cells invading the body 5.

Cancer cells grow rapidly, and chemotherapy drugs impede or slow tumor growth by arresting cell division and ultimately killing cancer cells 6, 7. However, these drugs are not selective, and normal cells and tissues, particularly those undergoing constant turnover throughout life, are also killed or impaired by chemotherapy 8. Cisplatin is a widely used platinum-based chemotherapeutic drug to treat various cancers, including testicular, ovarian, pancreatic, cervical, head and neck, and lung tumors 9-11. Unfortunately, up to 77% of cancer patients experience varying degrees of taste dysfunction after cisplatin treatment, which severely affects their nutrition intake. Therefore, finding effective therapies to maintain normal taste function is critical for improving the quality of life for cancer patients.

Cancer patients may experience various aspects of taste dysfunction, such as abnormal neural activity, mucosal inflammation, lack of zinc, and damage of chemosensory receptor cells 12. Oral mucositis is also a common and painful adverse reaction caused by chemotherapy. Tissue inflammation surrounding taste buds may cooperate with chemotherapy drugs to inhibit the proliferation and differentiation of taste stem/progenitor cells, thus leading to taste disorders. In clinic practice, some adjuvant drugs have been used to treat chemotherapy-induced taste disorders. Amifostine is a commonly used cytoprotective agent to reduce mucosal inflammation caused by chemotherapy 13. Previous studies have shown that irradiation causes loss of taste bud cells by disrupting taste cell renewal due to cell cycle arrest in taste progenitors 14. Conversely, activation of Wnt/β-catenin signaling has been found to alleviate radiation injury in taste cells 15. Vismodegib, a hedgehog pathway inhibitor used to treat basal-cell carcinoma, has been shown to reduce the numbers of PLCβ2- and Gustducin-expressing cells in taste buds 16. Similarly, deletion of Smoothened (Smo), a core signal transduction component of hedgehog signaling, mimics the effect of cancer drug sonidegib on taste organ morphology 17. However, the exact cellular and molecular mechanisms underlying taste disorder caused by chemotherapy drugs are still remain elusive.

Taste buds are primarily located on the fungiform (FF) papilla in the anterior tongue and the foliate (FL) and circumvallate (CV) papillae in the posterior tongue 18. We and the other group identified that Lgr5 (leucine-rich repeat-containing G-protein coupled receptor 5) marks adult taste stem/progenitor cells. Lgr5-expressing cells give rise to all types of taste cells in the posterior tongue 19, 20. According to the current working model for taste cell generation, Lgr5-expressing stem/progenitor cells differentiate into Sonic hedgehog (Shh)-expressing precursor cells (also known as type IV cells, or basal cells), which further differentiate into three major types of mature taste cells 20, 21. Type I taste cells are supporting cells, with NTPDase II and GLAST expressed on cell membrane surface 22. Type II cells are taste receptor cells sensing bitter, sweet and umami stimuli, marked by Gustducin, Trpm5, PLCβ2 and T1R3 23. Type III cells are presynaptic cells that mediate sour taste through ion channels, and marked by NCAM, Snap-25, Car4, and 5-HT 24.

Our study investigated the impact of cisplatin on taste cell homeostasis and taste function using animal and organoid models. We found that cisplatin had inhibitory effects on taste cell proliferation and taste receptor cell generation, with transcriptional changes associated with cell division, metabolic process, and inflammatory response observed in the circumvallate papilla. In the taste organoids, we found that LY411575, a Notch signaling inhibitor, restored taste cell proliferation, inhibited apoptosis, and promoted taste receptor cell generation that were reversely regulated by cisplatin. This was similar to the effect of cytoprotective agent amifostine. Additionally, LY411575 and amifostine were able to offset the increasing number of Pax1^+^ or Pycr1^+^ cells induced by cisplatin in the circumvallate papilla. Overall, our study sheds light on explaining potential mechanism underlying cisplatin-induced taste disorders, and identifies promising therapeutic agent and targets.

## Materials and Methods

### Animals

Wide type C57BL/6J mice were purchased from Jackson Laboratory and Shanghai Model Organisms. Genetically targeted heterozygous Lgr5-EGFP-IRES-CreERT2 mice (Stock number 008875; harboring a “knock-in” allele that abolishes Lgr5 gene function and expresses EGFP and CreERT2 fusion protein from the Lgr5 promoter/enhancer elements) were purchased from the Jackson Laboratory. Genotypes were confirmed using primer sets recommended by Jackson Laboratory. Male mice used for experiments were 8 to 12 weeks old, 20 to 25 g weight. Mice were housed with a 12/12 h light and dark cycles and free access to standard mice chow and water, except during gustatory behavior tests. The procedures of animal handling and tissue harvesting were approved by the institutional animal care and use committee (Permit Number: SYXK2022-0011).

### Gustometer assay

Gustometer tests were conducted using Davis MS-160 mouse gustometer (Dilog Instruments, Tallahassee, FL). We followed the standard procedure developed by several laboratories for testing mice 25, 26. Before testing of responses to taste solutions, mice were subjected to a few days of water training to familiarize with the gustometer. To motivate mice to lick from the sipper tube, they were water-deprived for 22.5 h prior to the training sessions. Each training session lasted 30 min. Immediately after each training session, mice were given 1 h of ad libitum access to water.

After water training, mice were given 3 injections of cisplatin (i.p., 7.5 mg/kg, one dose per three weeks). The day of the last cisplatin injection was set as day 1. Taste tests were performed on the days as described. The order of the taste stimuli was determined by a computer software that randomly selected the taste stimuli and a water tube. We tested three concentrations of each taste compound. For testing NaCl, mice were water-deprived for 22.5 h prior to the test. After the tests, mice were given free access to water and food for 1 h. For testing saccharin and sucrose, mice were given 1.5 mL of water and 1 g of food for 22.5 h. After tests, mice were given free access to food and water. For all tests, thirty-six 5 s trials were included, and taste solutions and water were randomly presented to mice according to computer generated random sequences. Tastant to water lick ratios were calculated by dividing the number of licks of a taste solution by the number of licks of water. However, this gustometer did not have apparatus to control olfactory cues. So, we cannot rule out the effect of olfactory cues.

### Gustatory nerve recording

Whole nerve taste responses from chorda tympani nerve were recorded according to previously described 27. Briefly, mice were deeply anesthetized with mixed anesthetics (10 mL/kg) consisted of ketamine (4.28 mg/mL), xylazine (0.86 mg/mL) and acepromazine (0.14 mg/mL). The trachea of mice was intubated and the pterygoid muscle were removed. Then the chorda tympani nerve was exposed and successfully dissociated by using a mandibular approach. The nerve was desheathed and positioned on a platinum electrode while the second electrode was placed in surrounding muscle and served as ground. Solutions were applied from one side of the tongue, using a syringe and allowed to remain on the tongue for ~30 s, and then rinsed with distilled water for ≥ 1 min. Solutions used for chemical stimuli were as follows: sucrose, saccharin, NaCl, HCl, QHCl and Monopotassium Glumate. The mean height of the tonic response was measured after 30 s of stimulus application, 0.1 M NH4Cl was applied periodically and the reaction value was used as the standard value. The mice were sacrificed by overdose of anesthesia after experiment.

### Chemical treatment

For all experiment except for gustometer assay and nerve recording, one dose of cisplatin at 7.5 mg/kg was injected intraperitoneally. Animals were sacrificed at Day 1, 4, 7, 14 post cisplatin injection. The rationale to select these four points is to determine the immediate effect (Day 1), the effect during damage-recovery process (Day 4 and 7) and when the regeneration was complete (Day 14). For gustatory nerve recording, mice received one dose of cisplatin every week (Cisplatin-S, three doses in three weeks) or one dose every three weeks (Cisplatin-L, three doses in nine weeks).

For cell protection test, 10 mg/kg LY411575 or 100 mg/kg amifostine was injected intraperitoneally 30 min before cisplatin treatment. All chemicals were purchased from Sigma Aldrich.

### Immunohistochemistry

Mice were deeply anesthetized with intraperitoneal injection of ketamine-xylazine (200 and 15 mg/kg body weight) before decapitation. The circumvallate tissue were dissected from tongue and fixed in 4% paraformaldehyde (Sigma Aldrich) overnight at 4 °C. The fixed tissue was then infiltrated with a series of sucrose solutions before being embedded in OCT. The frozen tissues were cut into 20 μm sections on a cryostat (Leica CM1950). After rinsing with PBS, the tissue sections were blocked for 1 h in 0.3% Triton X-100 in phosphate-buffered saline with 5% bovine serum albumin, and then incubated overnight at 4 °C with the primary antibodies, followed by incubation with secondary antibodies for 1 h at room temperature. The primary and secondary antibodies used were listed in Table S1. Mouse Alexa488 Zenon antibody labeling kit (Invitrogen) was used to label the mouse anti-Ki67 antibody as described previously 28. Tissues were mounted in Vectashield (Vector Laboratories). Fluorescent images were taken under a SP5/Leica confocal microscope with LAS AF Lite software.

### RNA-Seq analysis

RNA-Seq analysis was conducted by Novelbio Co. (Shanghai, China). Sequencing reads were mapped to the mouse genome using HISAT2. Transcriptome from RNA-Seq reads was reconstructed by StringTie. DESeq2 was used to evaluate expression differences, and Pearson's coefficient was calculated to determine the correlation among different groups. To cluster the global gene expression patterns in different samples, K-means clustering algorithm by RSEM software was used. Gene Ontology (GO, http://www.geneontology.org/) and Kyoto Encyclopedia of Genes and Genomes (KEGG) pathway analysis (http://www.genome.jp/kegg/) were performed. All the sequence data was analyzed on Novelbio Cloud Platform, and deposited on NCBI Sequence Read Archive (BioProject ID: PRJNA901384).

### Quantitative real-time PCR

Total RNA was extracted from the circumvallate papillae tissues of control and cisplatin-injected mice at Day 1, 4, 7, 14 post cisplatin injection, or circumvallate papillae tissues of mice treated with cisplatin, cisplatin/LY411575, cisplatin/amifostine and corresponding organoids using the E.Z.N.A. Total RNA Kit I (catalog #R6834-02, Omega) according to the manufacturer's instructions. The extracted RNA was dissolved in RNase-free water, and the purity and concentration were determined using a BioPhotometer (Metash). First-strand cDNA was synthesized using a PrimeScript RT Master Mix (catalog #RR036A, Takara). Primers used in this study were synthesized by Shanghai Tsingke Biotechnology Co., Ltd. Quantitative real-time PCR was performed on an Analytik Jena Real-Time PCR System. The reaction mixtures included a cDNA template, 0.2 mM primers, SYBR qPCR SuperMix (catalog #E096-01B, Novoprotein), and double distilled H_2_O. The relative expression levels were calculated using the 2^-ΔΔCt^ method. Primer sequences used were listed in Table S2.

### Taste organoid culture and chemical treatment

Tongues from 3-month-old age C57BL/6J mice were harvested from and injected with an enzyme mixture containing collagenase (1 mg/mL, Roche) and dispase (2 mg/mL, Roche) in Tyrode's solution (145 mM NaCl, 5 mM KCl, 5 mM NaHCO3, 10 mM pyruvate, 10 mM glucose, 10 mM HEPES) for 15~20 min at 37 °C. The circumvallate papillae areas were then dissected, cut into small pieces with scissors, and digested with 0.25% trypsin-EDTA (0.5~1 mL) for 15~20 min at 37 °C. Trypsinization was stopped by adding an equal volume of DTI. Samples were centrifuged at 1200 rpm for 3 min and supernatant was discarded. The tissues were mechanically dissociated into single cells using a 1 mL firepolished syringe needle. Single cell suspension was filtered through a 70-μm and 40-μm nylon mesh cell strainers (BD Falcon).

The single cell suspensions were seeded at 1000 cells/well in an Ultra-low-attached 24-well plate and cultured in taste organoid culture medium. The culture medium was based on DMEM/F12 medium (Life Technologies no. 11320033), supplemented with R-spondin-1 (200 ng/mL, R&D, #4645RS025), Noggin (100 ng/mL, R&D, #6057NG025), B27 (2% (vol/vol), ThermoFisher, #17504044), epidermal growth factor (50 ng/mL, Peprotech, #315-09), N2 (1%, ThermoFisher, #17502-048), penicillin-streptomycin (100×, ThermoFisher, #15140122), Glutamax (1%, ThermoFisher, #35050061), HEPES (1 mM, ThermoFisher, #15630080), Y-27632 (10 μM, Sigma Aldrich, #Y0503) and 3% chilled Matrigel (BD Biosciences, #356231). The medium was changed every three to five days based on the density of growing organoids, while organoid density was generally similar across wells. Organoids were maintained at 37 °C with 5% CO2. The organoids were passaged every 10~14 days. When passaging, organoids were collected by centrifugation at 1,200 rpm for 3 min, followed by incubation in 0.25% trypsin-EDTA at 37 °C for 10 min. Single cell suspension was made by drawing through 1 mL-microsyringe and cells were seeded in taste organoid culture medium at density of 500 single cells per well in Ultra-low-attached 24-well plates (Corning).

Organoids were treated with cisplatin at concentrations of 5, 10, 15 μM. Short-term treatments were administered by adding cisplatin on Day 3 and withdrawing on Day 5. Long-term treatments were administered by adding cisplatin on Day 4 and withdrawing on Day 12. To assess the cytoprotective effect, 10 µM LY411575 or 10 µM amifostine was added 30 min before cisplatin treatment.

### Immunostaining for taste organoid

Organoids were collected and fixed in 4% paraformaldehyde for 15 min at room temperature and washed with phosphate-buffered saline (PBS) for three times. Then organoids were blocked with 5% bovine serum albumin (BSA) with 0.3% Triton X-100 in PBS for 1 h at room temperature, and incubated with primary antibodies overnight in a humidified chamber at 4 °C. After washed with PBS, the organoids were incubated with secondary antibodies at room temperature for 2 h. DAPI (ThermoFisher, #D3571) was used to counterstain the nuclei. The primary and secondary antibodies used were listed in Table S1. Organoids were then mounted in Vectashield (Vector Laboratories). Fluorescent images were captured using a SP5/Leica confocal microscope with LAS AF Lite software.

### Luminex liquid suspension biochip detection

Inflammatory cytokines and chemokines Luminex liquid suspension biochip detection was performed by Wayen Biotechnologies (Shanghai, China). The circumvallate papillae tissues were harvested from mice receiving saline or cisplatin at Day 1 and Day 4 post treatment, and washed in PBS to remove debris. The tissues were fully ground in RIPA solution, and the supernatant was collected by centrifuge. Protein concentrations were determined by the BCA Protein Quantification Kit (Yeasen, 20201ES90), according to standard procedures. The inflammatory cytokines and chemokines detection was performed using a Bio-Plex Pro Mouse Cytokine Grp I Panel 23-plex kit (Wayen Biotechnologies, Shanghai) by Luminex 200 system (Luminex Corporation, Austin, TX, USA) according to the manual. Briefly, 50 µL standard, experimental and blank group samples were added into microbeads-treated 96-well plates. Plates were then oscillated on a flat plate vibrator at 850 rpm and incubated in the dark at room temperature for 30 min. All samples were discarded, and 25 µL diluted Detection Antibody were added into each well, and the plate was incubated at room temperature for 30 min. After removal of antibody, diluted Streptavidin-PE was added in plates and incubated for 10 min. After wash, microbeads in wells were suspended by adding 125 µL Assay Buffer. The data were measured and collected by the Luminex 200 system.

### Western blot

Proteins were extracted from taste organoids treated with cisplatin, cisplatin/LY411575 or cisplatin/amifostine using a Total Protein Extracion Kit (Comiike; CAT#PE1202). The sample were prepared with boiling in 5× SDS Loading Buffer for 5 min. SDS-PAGE was used to separate total protein, and 3 μg sample was loaded into each well. The proteins were transferred to PVDF membrane and the membrane was blocked in 5% non-fat milk for 1 h. The following primary antibodies were used to detect protein expression: cleaved Caspase 3 (#9661; CST, 1:500), Pax1 (#ab252847; Abcam, 1:500), Pycr1 (#ab102601, Abcam, 1:1000), and β-actin (ab8226, Abcam, 1:2000). After incubation in primary antibodies at 4 °C overnight, membrane was incubated with secondary HRP antibodies at room temperature for 1 h. Protein bands were visualized using ECL system (ThermoFisher).

### Quantitative analysis

Each group contained 9 mice in behavioral and nerve recording experiments, 3 mice in tissue immunostaining and RNA-Seq analysis, 3 independent experiments for qPCR analysis, cytokine assay, and organoid staining. The image acquisition settings were consistent across all treatments. The number of positively stained cells and DAPI^+^ cells were quantified per taste bud or organoid using Image J software by analyzing immunostaining pixels. The percentage of positively stained cells was calculated as [number of positively stained cells] / [number of DAPI^+^ cells]. The organoids size was determined by SPOT 5.1 Advanced Software based on two-dimensional images. Statistical analyses were conducted using GraphPad Prism software (GraphPad Software, La Jolla, CA). Results are expressed as mean ± standard deviation (S.D.), and statistical significance was determined by using an unpaired t test, one-way ANOVA with Dunnett's multiple-comparisons test or two-way ANOVA with Sidak's comparisons test. A p-value of < 0.05 was considered statistically significant.

## Results

### Chemotherapy drugs attenuate taste tissue homeostasis and taste function *in vivo*

**Figure 1:**
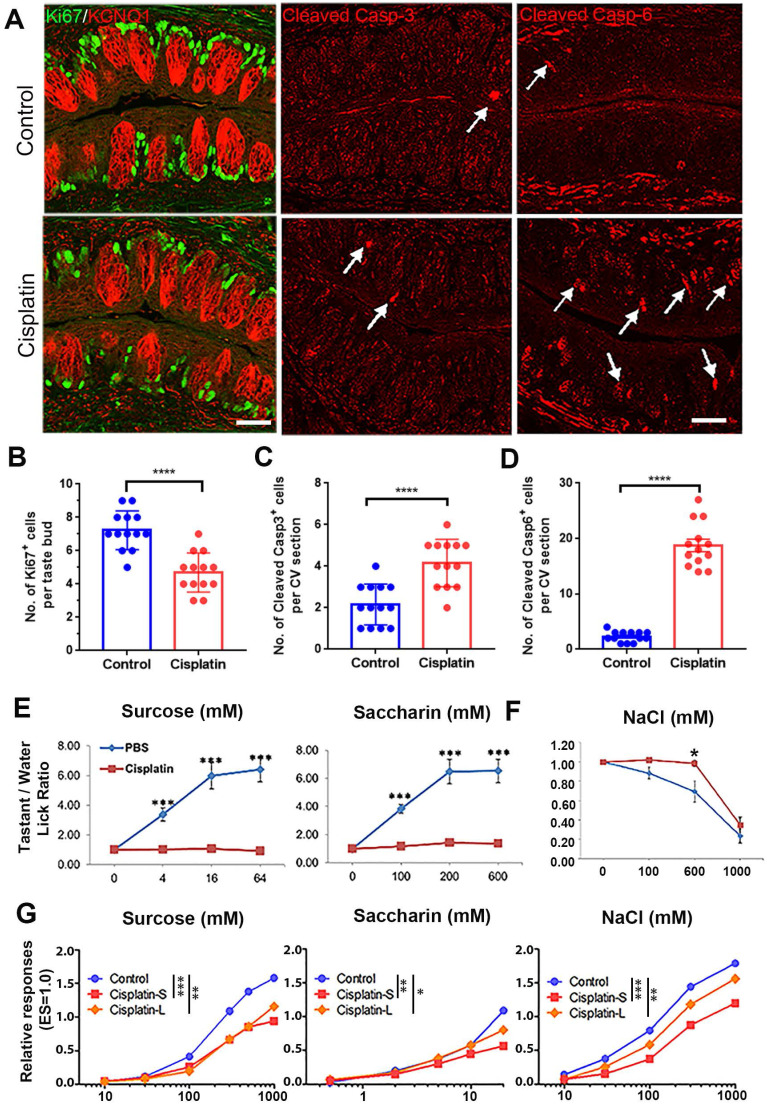
Cisplatin weakens proliferation, induces apoptosis in taste circumvallate papilla, and attenuates behavioral responses and taste nerve responses. (A) Immunostaining of Ki67 and KCNQ1, cleaved Caspase 3, cleaved Caspase 6 in circumvallate sections from control mice (PBS) and mice injected with cisplatin (7.5 mg/kg). Tissues were collected at Day 3 after a single injection of PBS or cisplatin. (B-D) Quantification of Ki67^+^ (B), cleaved Caspase 3^+^ (C), cleaved Caspase 6^+^ cells (D) in the circumvallate papilla collected at Day 3. **** p < 0.0001, by unpaired t test. (E, F) Behavioral responses of mice treated with cisplatin toward sweet-tasting compounds sucrose and saccharin (E), salty-tasting compound NaCl (F). n = 9 wild-type C57BL/6 mice per treatment. These groups of mice were tested at Day 5 after receiving injection of cisplatin (7.5 mg/kg) or PBS (control). PBS-treated and cisplatin-treated mice were tested side by side to reduce procedure-related variability. * p < 0.05, *** p < 0.001, by unpaired t test. (G) Whole-nerve recordings from chorda tympani nerves of mice with or without cisplatin treatment. Mice received three doses of cisplatin or saline treatment via intraperitoneal injections either one dose every week (Cisplatin-S) or one dose every three weeks (Cisplatin-L). Data were integrated chorda tympani responses of control or treated wild-type mice in response to lingual application of a concentration series of sucrose, saccharin or NaCl, normalized to the response to electric stimulation (ES = 1.0). * p < 0.05, ** p < 0.01, *** p < 0.01, by two-way ANOVA with Sidak's comparisons test. Scale bars, 25 μm.

We investigated the effects of chemotherapy drugs on taste cell proliferation, apoptosis, and taste behavior in mice. We administered cisplatin (Cis, 7.5 mg/kg), 5-fluorouracil, (5-FU, 150 mg/kg), and paclitaxel (PTX, 115 mg/kg) to mice at dosages within the range used for cancer treatment 29, and examined the changes in the number of Ki67^+^ proliferative cells and cleaved Caspase 3/6^+^ apoptotic cells in the circumvallate papilla at Day 3 post injection. Our results showed that all three drugs reduced the number of Ki67^+^ cells and increased the number of cleaved Caspase 3 (Casp-3) or cleaved Caspase 6 (Casp-6) cells (Figure 1A-D, Figure S1A-C). The number of Ki67^+^ cells was reduced by 35±5% (p < 0.0001), 50 ± 4% (p < 0.0001), or 47 ± 5% (p < 0.0001) with injection of cisplatin, 5-FU or PTX at Day 3, compared to control group receiving saline (Figure 1B, Figure S1B). In addition, the number of cleaved Casp-3^+^ cells per CV section was significantly increased by 93 ± 15% (p < 0.0001), 174 ± 14% (p < 0.0001) or 134 ± 17% (p < 0.0001) with the treatment of cisplatin, 5-FU or PTX (Figure 1C, Figure S1C), while the number of cleaved Casp-6^+^ cells was increased by 716 ± 49% (p < 0.0001) with cisplatin injection (Figure 1D). These results indicate that all three chemotherapy drugs may arrest or slow the cycling of taste proliferative cells and induce apoptosis in taste buds.

To answer whether chemotherapy alters taste behavior, we performed brief-access gustometer assay to determine how mice respond to tastants after chemotherapy. The gustometer data suggested that responses to sweet and salty tastants were altered by cisplatin (Figure 1E-F). Mice showed reduced preferences for sweet-tasting compounds saccharin and sucrose at Day 5 after receiving cisplatin injection (Figure 1E, p < 0.001 for saccharin, p < 0.001 for sucrose). Besides, cisplatin treatment reduced avoidance of high salt. For mice treated with cisplatin, no clear avoidance was observed toward 600 mM NaCl, which was apparently avoided by mice receiving PBS, suggesting that cisplatin weakens avoidance to high salt taste (Figure 1F, p < 0.05).

To address whether behavioral alteration by cisplatin was directly associated with taste cells, we conducted gustatory nerve recordings to assess the activity of taste cells. In order to compare the long/low effect to the short/high effect of cisplatin, mice were injected three doses of cisplatin totally either one dose every week (Cisplatin-S) or one dose every three weeks (Cisplatin-L). Results showed that both injection schedules led to significant reduction in taste nerve responses toward all tastants (Figure 1G, Figure S1D-E), supporting that reduced chorda tympani nerve responses may contribute to impaired taste behavior after chemotherapy. Collectively, these data demonstrate that chemotherapy disrupts taste cell homeostasis and function.

### Cisplatin inhibits taste cell generation in the circumvallate papilla

**Figure 2:**
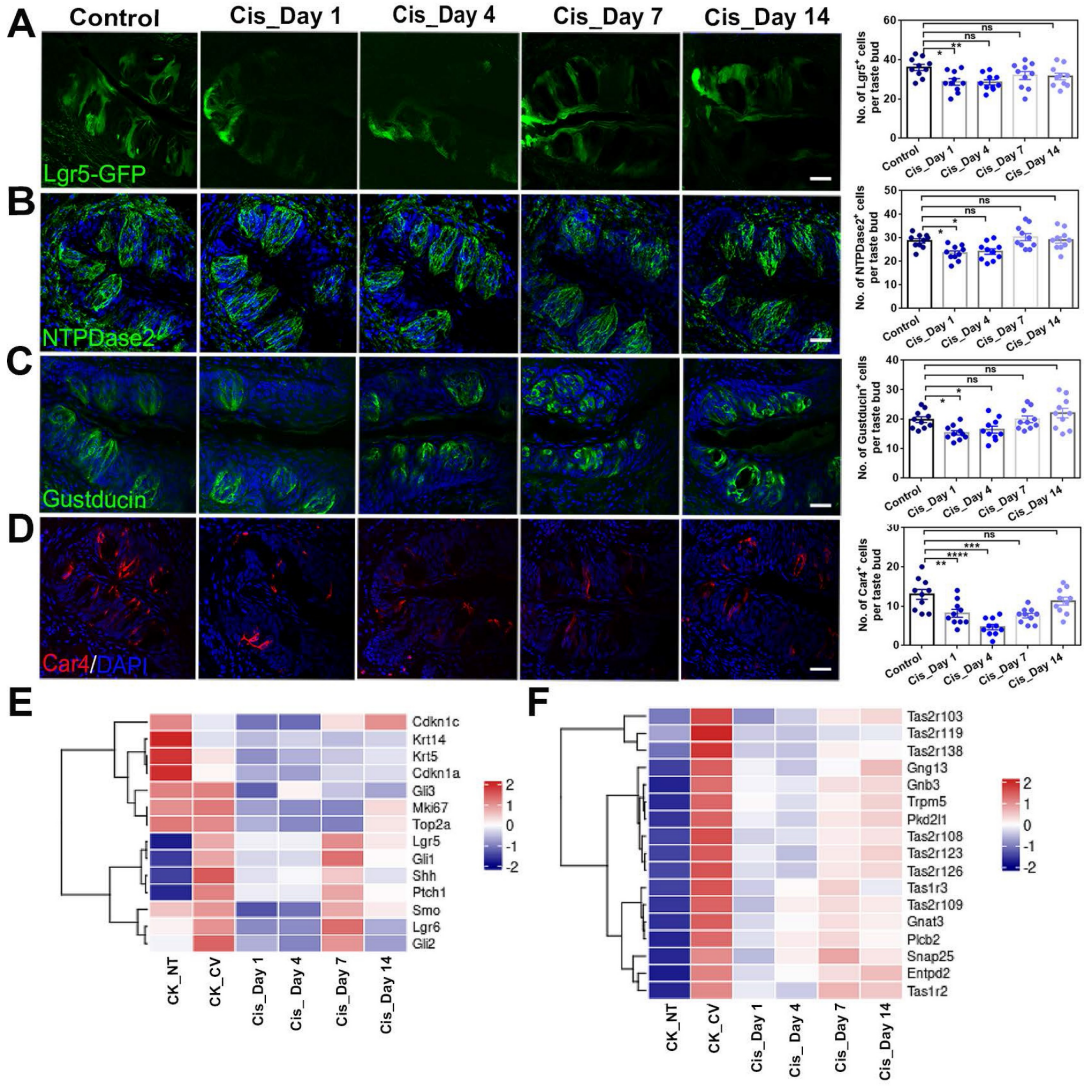
Cisplatin treatment impairs taste progenitors and receptor cells in the circumvallate papilla. (A-D) Confocal images and statistical analysis of Lgr5-EGFP^+^ (A), NTPDase 2^+^ (B), Gustducin^+^ (C), Car4^+^ (D) cells in the circumvallate papilla of Lgr5-GFP-Cre^ERT2^ mice, injected with one dose of cisplatin and sacrificed at Day 1, 4, 7 and 14 after injection. ns, not significant, *p < 0.05, ** p < 0.01, *** p < 0.001, **** p < 0.0001, by one-way ANOVA with Dunnett's multiple-comparisons test. (E, F) Heatmap showing the expression of taste progenitor and proliferative cell markers (E), taste receptors and taste transduction genes (F) in the non-taste (NT) tissue, as well as circumvallate papillae of saline control and cisplatin-treated mice sacrificed at Day 1, 4, 7 or 14 after treatment. Scale bars, 25 µm.

To investigate whether cisplatin treatment influenced taste progenitor cells, Lgr5-EGFP-Cre^ERT2^ mice were used and sacrificed at different time points (Day 1, 4, 7 and 14) after cisplatin exposure. In the tongue, Lgr5 marks taste stem/progenitor cells 19, 30. Our results showed that the number of Lgr5-EGFP^+^ cells per taste bud in the circumvallate papilla was decreased by 21±5% (p < 0.05) and 21 ± 4% (p < 0.01) at Day 1 and Day 4 after cisplatin treatment (Figure 2A), respectively, suggesting that chemotherapy affects the homeostasis of Lgr5^+^ stem/progenitor cells in the taste bud. To further investigate the impact of cisplatin on the taste cell generation, we immunostained circumvallate papilla sections using antibodies against NTPDase2 (a marker for type I taste cells), Gustducin (a marker for type II taste cells) and Car4 (a marker for type III taste cells). Our results showed that cisplatin treatment significantly reduced the number of mature taste cells, particularly on Day 1 and Day 4 after chemotherapy treatment (Figure 2B-D). Compared to the saline controls, the number of NTPDase2^+^ or Gustducin^+^ cells in the circumvallate papilla from cisplatin-treated animals were significantly reduced by 18±3% (p < 0.05) and 16 ± 4% (p < 0.05), or 23 ± 4% (p < 0.05) and 17 ± 6% (p > 0.05) on Day 1 and Day 4. However, the NTPDase2^+^ or Gustducin^+^ cell generation was recovered on Day 7 and Day 14 after cisplatin injection (Figure 2B-C). The number of Car4^+^ cells per CV section was reduced by 37±8% (p < 0.01), 64 ± 5% (p < 0.0001) and 42 ± 5% (p < 0.001) in cisplatin-treated groups on Day 1, 4 and 7, respectively, compared to the saline controls, but recovered on Day 14 after cisplatin treatment (Figure 2D).

To gain a deeper understanding of the transcriptional changes following cisplatin treatment, we conducted RNA-Seq analysis to examine the expression levels of taste cell and taste stem/progenitor markers after chemotherapy. As shown in Figure 2E, the expression levels of proliferating cell markers (Mki67 and Top2a), pan basal cell markers (Krt14 and Krt5), and taste stem/progenitor cell markers (Lgr5, Lgr6, Ptch1, Gli1 and Shh), were significantly reduced at Day 1 and Day 4 after cisplatin injection. The expression levels of taste receptors and taste transduction genes were also markedly decreased at Day 1 and Day 4 after cisplatin injection (Figure 2F). While expression levels of these genes were partially recovered from Day 7, they still remained lower than those observed in the saline-treated circumvallate papilla at Day 14 (Figure 2F). Therefore, we conclude that chemotherapy impairs the homeostasis of Lgr5^+^ progenitor cells and generation of taste receptor cells, broadly decreases the expression levels of taste stem/progenitor markers as well as taste receptor and transduction genes in the circumvallate papilla.

### Cisplatin leads to transcriptional alterations associated with inflammatory response and cell division

**Figure 3:**
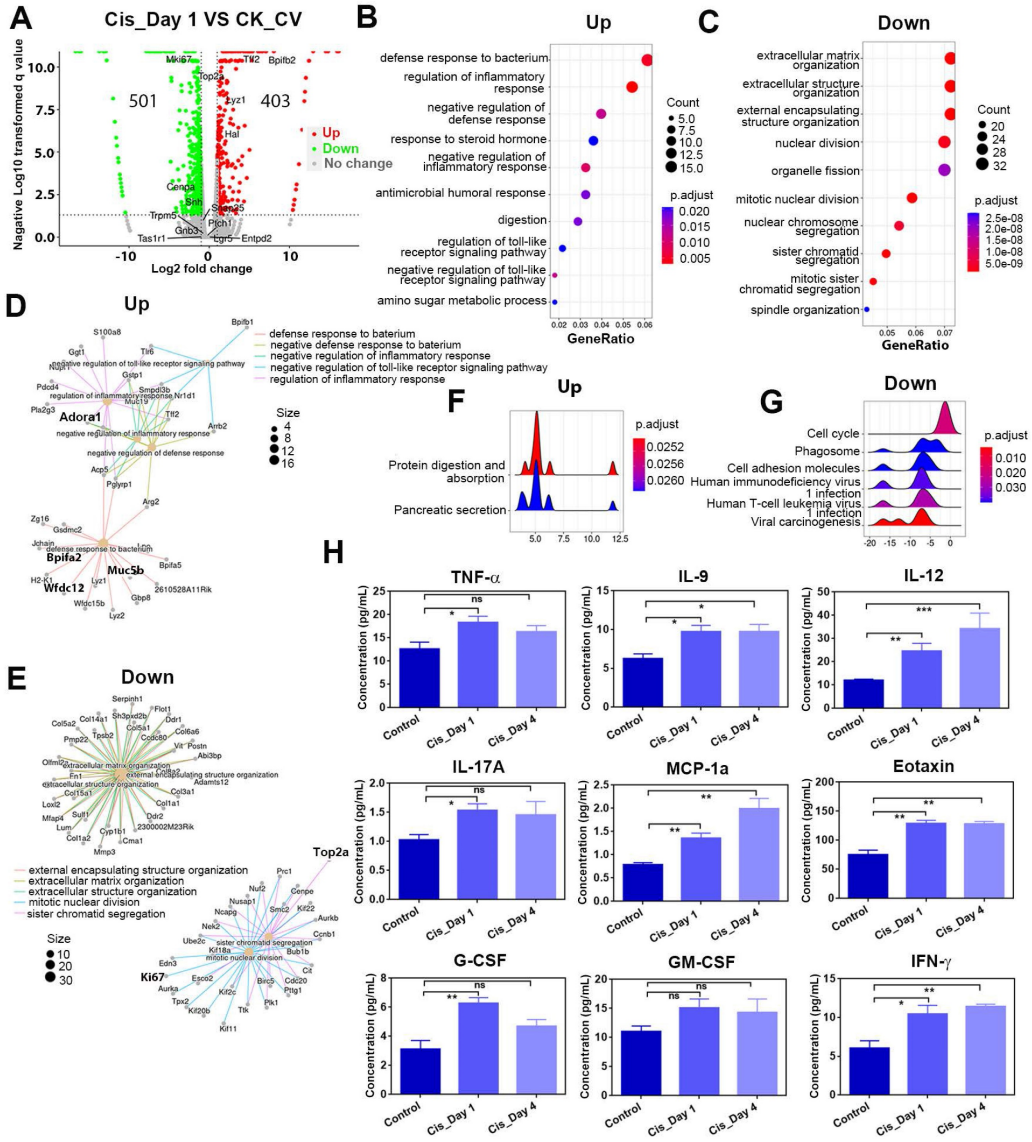
RNA-Seq analysis shows the transcriptional alteration related to inflammatory activation in the circumvallate papilla at Day 1 after cisplatin treatment. (A) Volcano plot showing the differentially expressed genes (DEGs) between saline- and cisplatin-treated circumvallate papillae at Day 1 post injection. (B, C) GO enrichment analysis of upregulated (B) and downregulated genes (C) between the circumvallate papillae from saline- and cisplatin-injected mice at Day 1 post treatment. (D, E) GO network analysis showing the critical genes involved in upregulated (D) and downregulated (E) GO terms. (F, G) Ridge plots of the GO terms of upregulated (F) and downregulated (G) genes, defined by the GSEA. X-axis was expression level (TPM). Peaks were colored by adjusted p-value per GO term. (H) Luminex liquid suspension biochip assay detected the concentration of inflammatory cytokines and chemokines in the circumvallate papilla of mice injected with saline or cisplatin at Day 1 and Day 4. ns, not significant, * p < 0.05, ** p < 0.01, *** p < 0.001, by one-way ANOVA with Dunnett's multiple-comparisons test.

As the most prominent changes were observed immediately after cisplatin injection, we analyzed the differentially expressed genes (DEGs) between circumvallate papillae from cisplatin-treated mice at Day 1 post-injection (Cis_Day 1) and control group injected with saline (CK_CV). The volcano plot displayed a substantial number of DEGs on Day 1 after cisplatin injection compared to the control group (Figure 3A).

**Figure 4:**
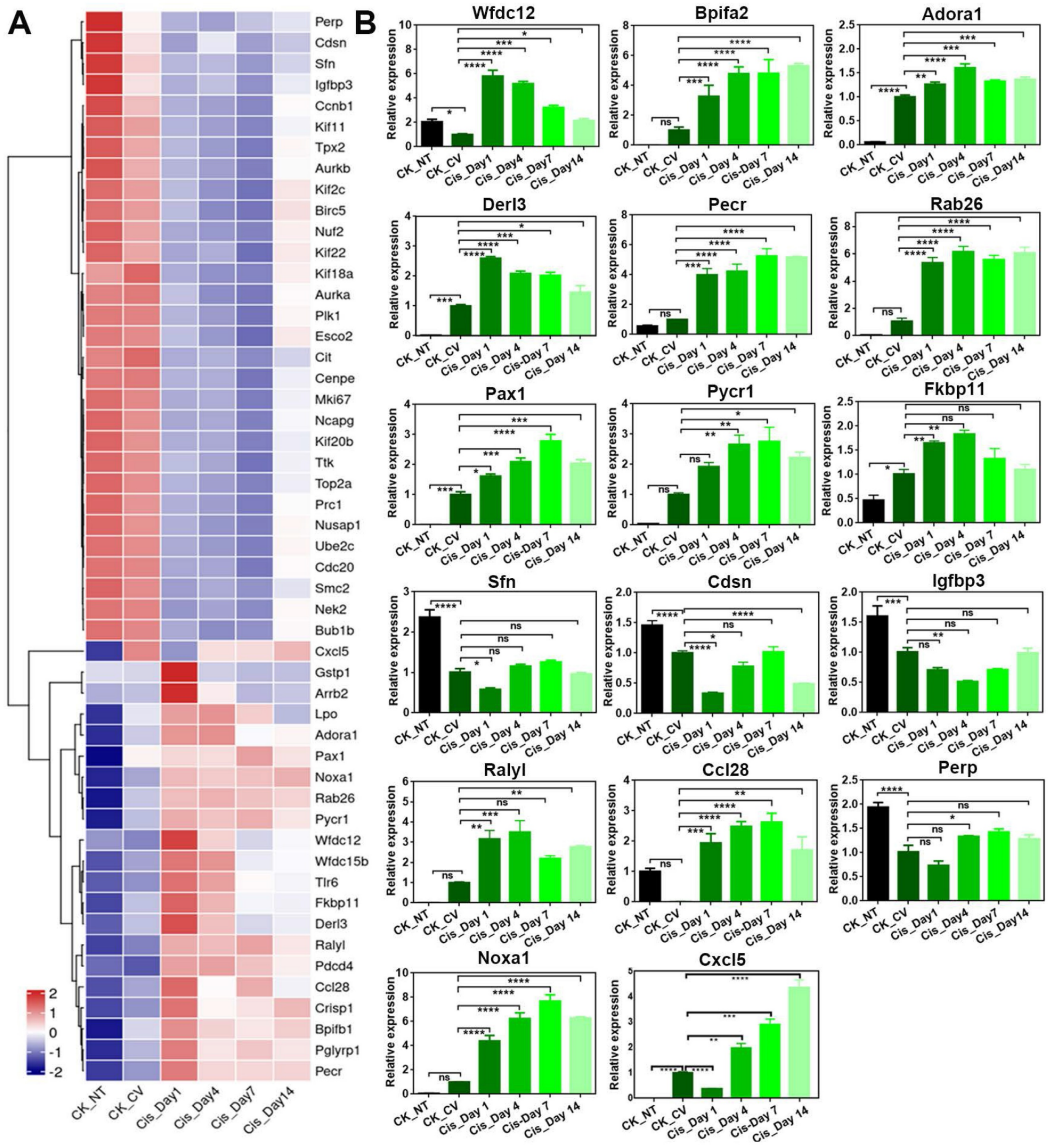
Cisplatin changes expression level of genes associated with multiple biological process in the circumvallate papilla. (A) Heatmap showing down- and up-regulated genes in the circumvallate papilla after cisplatin injection. (B) Quantitative PCR showing the change in expression level of genes selected from GO network, and genes associated with inflammatory response, metabolic response, and epidermal barrier, using circumvallate papillae from saline and cisplatin-injected mice at Day 1, 4, 7 and 14, as well as non-taste (NT) tissues. ns, not significant, * p < 0.05, ** p < 0.01, *** p < 0.001, **** p < 0.0001, by one-way ANOVA with Dunnett's multiple-comparisons test.

Gene Ontology (GO) enrichment analysis demonstrated that upregulated genes participated in defense response and regulation of inflammatory response, while downregulated genes were associated with extracellular matrix organization and nuclear division (Figure 3B-C). By GO network analysis, we identified crucial upregulated genes involved in regulating the inflammatory response and defense response to bacteria, including Wfdc12, Bpifa2, Adora1 (Figure 3D), which showed higher mRNA expression levels in cisplatin-injected groups compared to saline control group, as confirmed by quantitative PCR analysis (Figure 4B). A ridge plot of the GO terms defined by the Gene Set Enrichment Analysis (GSEA) indicated that gene set enrichment at the top of the ranked list, such as protein digestion and absorption, was significantly enriched in cisplatin treatment group (Figure 3F). Quantitative PCR analysis showed that expression levels of some metabolic genes, such as Derl3 and Pecr, were significantly upregulated in the circumvallate papilla following chemotherapy (Figure 4B). GO network disclosed the downregulated critical genes involved in extracellular matrix organization and nuclear division such as collagen genes, Top2a and Ki67 (Figure 3E), while GSEA showed the downregulated gene sets associated with cell cycle, phagosome, and cell adhesion molecules (Figure 3G). Thus, our findings suggest that cisplatin treatment results in significant transcriptional change in the circumvallate papilla, which affects multiple functions, including the regulation of inflammatory response, metabolic process, extracellular matrix organization, and nuclear division.

To further confirm that cisplatin triggers inflammatory activation, we used the Luminex liquid suspension chip to measure concentrations of various inflammatory cytokines and chemokines in circumvallate papilla of cisplatin- and saline-treated mice. Our results showed that the concentrations of proinflammatory cytokines, such as TNF-α, IL-9, IL-12, IL-17a, as well as chemokines such as MCP1a (Ccl2) and Eotaxin (Ccl11) were elevated in the circumvallate papilla following cisplatin treatment at Day 1 and Day 4 (Figure 3H). Furthermore, concentrations of critical modulators to inflammation and immunity, including G-CSF, GM-CSF, IFN-γ were also increased in the circumvallate papilla with cisplatin treatment (Figure 3H). These data provide strong evidence that cisplatin induces inflammatory activation in the circumvallate papilla.

Besides Bpifa2 and Wfdc12, other genes associated with inflammatory response such as Rab26 and Pax1 were confirmed to be upregulated after cisplatin treatment by RNA-Seq and quantitative PCR analysis (Figure 4A-B). Genes associated with apoptosis, such as Pycr1 and Fkbp11, were upregulated following cisplatin treatment (Figure 4A-B). This was consistent with the increasing number of cleaved Caspase 3^+^ and Caspase 6^+^ cells in the circumvallate papilla after cisplatin injection (Figure 1A, 1C-D). Moreover, the expression of Sfn, an important regulator in epidermal barrier, was found to be downregulated after chemotherapy (Figure 4A-B). This suggests the impairment in epidermal defense when the circumvallate papilla is exposed to cisplatin. Interestingly, we found that the expression levels of most genes showed apparent recovery at Day 14 post-cisplatin injection, as shown in Figure 4.

We then analyzed the DEGs between cisplatin groups at Day 14 (Cis_Day 14) and Day 7 (Cis_Day 7) (Figure S2A). GO enrichment analysis showed that upregulated genes were associated with nuclear division, while downregulated genes participated in metabolic process (Figure S2B-C). These results were contrary to GO enrichment analysis on the DEGs between cisplatin group at Day 1 and saline control (Figure 3B-C). Furthermore, GO network analysis indicated that a few G2/M phase related genes such as Top2a, Ube2c, Cdc20, Ccnb2 were involved in the upregulated processes (Figure S2D). Ridge plot of the upregulated GO terms indicated that gene set enrichment at the top of the ranked list including cell cycle was enriched in cisplatin group at Day 14 compared to Day 7 (Figure S2F). Meanwhile, downregulated genes including Cyp2a5 and Per2 were found to participate in metabolic process (Figure S2E). Ridge plot of the downregulated GO terms showed that downregulated metabolic pathway genes were enriched in cisplatin-treated circumvallate papilla at Day 14 compared to Day 7 (Figure S2G). Collectively, our RNA-Seq and qPCR data support that chemotherapy leads to massive transcriptional alterations involved in multiple biological functions such as inflammatory response, nuclear division and metabolic process in the taste bud.

### Cisplatin inhibits proliferation and taste cell generation in taste organoids

We then determined whether cisplatin affected cell proliferation and taste cell generation in *in vitro*-cultured taste organoids. We applied single dose of cisplatin treatment at three concentrations in taste organoids at different culture stages. Firstly, cisplatin was added at Day 0 post culture and kept for 5 days. We found the size of organoids was significantly reduced in the presence of 5, 10, 15 µM cisplatin (Figure S3A-B). Thus, cisplatin restrains growth when taste organoids were treated at the beginning of *in vitro* culture. Next, we asked if shorter period of cisplatin treatment, added at Day 3 when the organoid was formed and withdrawn at Day 5 post culture, could affect the taste organoid growth. With incubation for 48 h from Day 3, organoids showed apparent reduction in the size at Day 5, 6 and 8, respectively (Figure S3C-D). Therefore, shorter incubation of cisplatin, added when organoids are formed, attenuates organoid growth.

Then, we asked whether chemotherapy attenuated taste cell generation in organoids. Firstly, cisplatin was added at Day 4 when organoids were under immature state, and organoids were incubated with cisplatin for 8 days. Krt8 is a pan taste cell marker 30-32. The number of Krt8^+^ taste cells in each organoid was decreased by 44 ± 4% (p < 0.0001), 69 ± 3% (p < 0.0001), and 82 ± 3% (p < 0.0001) in the presence of 5, 10, and 15 µM cisplatin, respectively (Figure S3E-F). Meanwhile, the number of Car4^+^ Type III taste receptor cells per organoid was reduced by 50 ± 8% (p < 0.05), 72 ± 4% (p < 0.01), and 88 ± 3% (p < 0.001) when treated with 5, 10, and 15 µM cisplatin compared to saline-treated controls (Figure S3E-G).

Similarly, the percentages of NTPDase2^+^ Type I and PLCβ2^+^ Type II taste cells was significantly reduced by 77±3% (p = 0.0019) and 89±3% (p = 0.0003) with treatment of 5 µM cisplatin (Figure S4A-B). Thus, 8-day incubation of cisplatin added at immature state inhibits taste cell differentiation in circumvallate papilla-derived taste organoids. We also added cisplatin at Day 12 post culture when organoids were mature. After 2-day incubation, the number of Ki67^+^ proliferative cells per organoid was reduced by 70 ± 6% (p = 0.0008) in the presence of 5 µM cisplatin (Figure S4C-D), while the number of cleaved Caspase 3^+^ apoptotic cells per organoid was increased by 104 ± 16% (p = 0.0016) compared to saline controls (Figure S4E-F). We then determined whether inhibition of Caspase 3 counteracted the effect of cisplatin on taste organoids. Caspase 3 inhibitor was effective in preventing apoptosis and restoring cell proliferation, as evidenced by a 79 ± 4% decrease (p = 0.0001) in the percentage of cleaved Caspase 3^+^ cells and an increase by 157 ± 27% (p = 0.0031) in Ki67^+^ cells in the cisplatin-pretreated taste organoids compared to the non-Caspase3 inhibitor group (Figure S4C-F). Moreover, compared to cisplatin-treated organoids without Caspase 3 inhibitor treatment, inhibition of Caspase 3 promoted generation of taste cells, showing an increase by 27 ±9% (p = 0.0927), 336 ± 22% (p = 0.0008), and 569 ± 53% (p < 0.0001) in the percentage of Krt8^+^, PLCβ2^+^, Car4^+^ taste cells (Figure S4D-E, S4G-H). These data suggest that 2-day incubation of cisplatin in mature taste organoids inhibits cell proliferation, promotes apoptosis, and restrains taste cell generation, while inhibition of Caspase 3 offsets the effect of cisplatin.

### LY411575 alleviates the inhibitory effect of cisplatin on taste cell proliferation and differentiation

**Figure 5:**
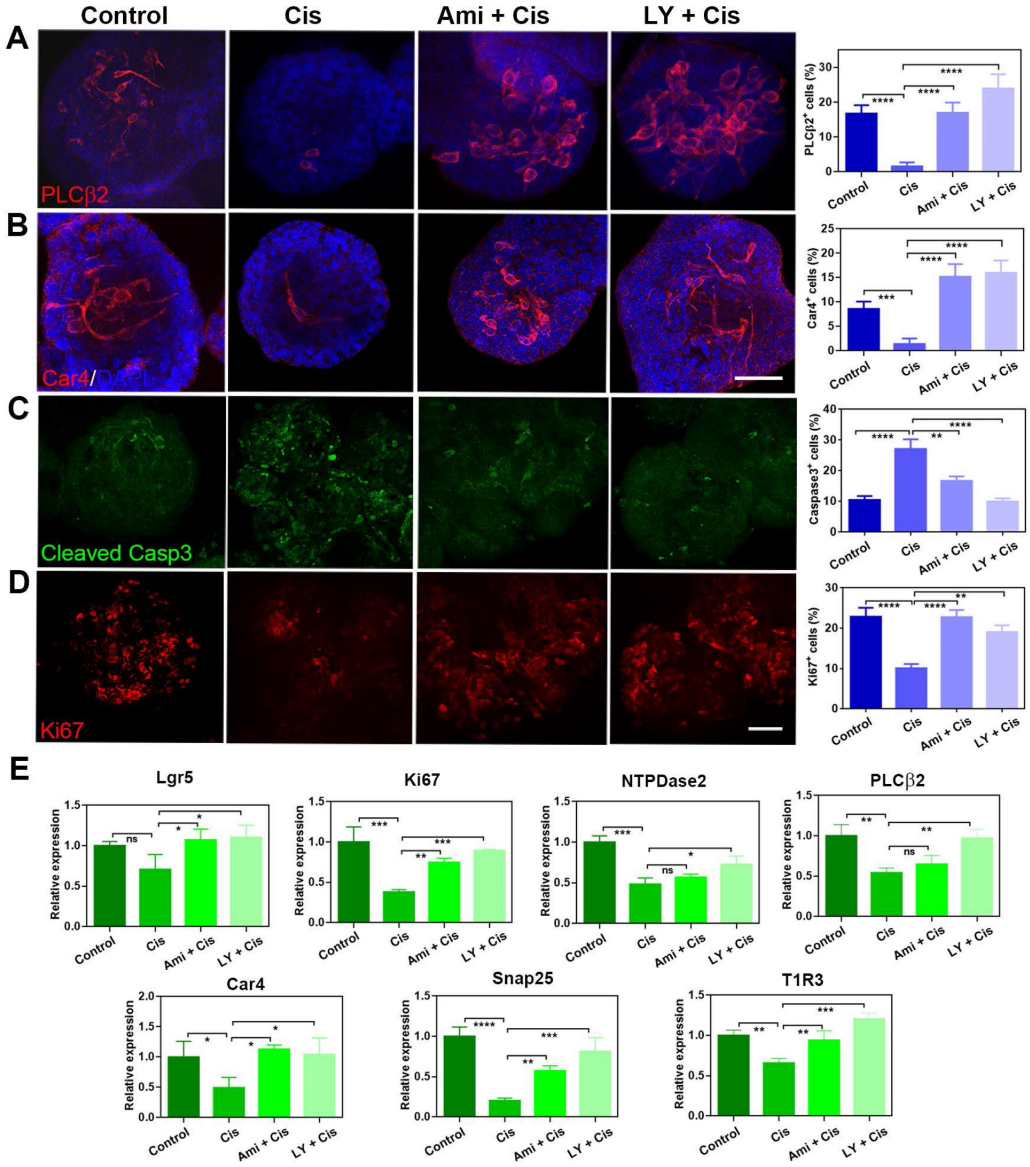
LY411575 counteracts the effect of cisplatin in taste organoids. (A, B) Confocal images and quantification of PLCβ2^+^ (A), Car4^+^ (B), cleaved Caspase 3^+^ cells (C), Ki67^+^ cells (D) in taste organoids treated with saline, cisplatin, cisplatin/amifostine, cisplatin/LY411575. ** p < 0.01, *** p < 0.001, **** p < 0.0001, by one-way ANOVA with Dunnett's multiple-comparisons test. (E) Quantitative PCR showing expression levels of Lgr5, Ki67, NTPDase2, PLCβ2, Car4, Snap25, T1R3 in taste organoids treated with saline, cisplatin, cisplatin/amifostine, cisplatin/ LY411575. ns, not significant, * p < 0.05, ** p < 0.01, *** p < 0.001, **** p < 0.0001, by one-way ANOVA with Dunnett's multiple-comparisons test. Scale bars, 50 µm.

Our previous work demonstrated that LY411575 (a γ-secretase and Notch inhibitor) promoted taste cell generation in circumvallate papilla-derived taste organoids 33. We thus explored whether this chemical could alleviate the inhibitory effect of cisplatin on cell proliferation and taste cell generation in organoids. In the presence of LY411575, the generation of taste receptor cells in cisplatin-treated organoids was recovered. The percentages of PLCβ2^+^ type II cells and Car4^+^ type III cells per organoid were significantly decreased by 81 ± 6% (p < 0.0001) and 84 ± 6% (p < 0.0001) with cisplatin treatment (Figure 5A-B). By comparison, co-application with LY411575 increased the percentages of PLCβ2^+^ cells and Car4^+^ cells by 1400 ± 113% (p < 0.0001) and 1043±81% (p < 0.0001) compared to cisplatin-treated group, and the percentage of Car4^+^ or PLCβ2^+^ cells in LY411575/cisplatin group was comparable to those in non-injected control group (Figure 5A-B). The effect of LY411575 was similar with cytoprotective adjuvant amifostine, since co-application of amifostine increased the percentages of PLCβ2^+^ cells and Car4^+^ cells by 962 ± 81% (p < 0.0001) and 986±83% (p < 0.0001) than the cisplatin-treated group (Figure 5A-B). Besides, the percentage of cleaved Caspase 3^+^ cells per organoid was increased by 158 ± 30% (p < 0.0001) with cisplatin treatment (Figure 5C), while the ratio of Ki67^+^ proliferative cells was significantly decreased by 56 ± 5% (p < 0.0001) in cisplatin-treated organoids compared to saline controls (Figure 5D). Co-application with LY411575 showed reduction in the percentage of cleaved Caspase 3^+^ cells by 63 ± 4% (p < 0.0001, Figure 5D) and increase in the ratio of Ki67^+^ cells per organoid by 124 ± 6% compared to cisplatin group (p < 0.001, Figure 5C). Furthermore, the cytoprotection of LY411575 against cisplatin showed dose-dependent effect, with 22±6% (p > 0.05), 61 ± 5% (p < 0.0001), and 80±8% (p < 0.0001) reduction in the percentage of cleaved Caspase 3^+^ apoptotic cells per cisplatin-treated organoids with treatment of LY411575 at 1 μM, 5 μM and 10 μM (Figure S5A-B). Cytoprotective adjuvant amifostine showed the similar effects on cisplatin-treated organoids as LY411575. The number of Ki67^+^ cells was increased by 87 ± 9% (p < 0.0001, Figure 5C), while the number of cleaved Caspase 3^+^ cells was decreased by 38 ± 5% in amifostine/cisplatin-treated organoids in contrast to cisplatin-treated group (p < 0.01, Figure 5D). The cleaved Caspase 3 expression change at protein level was measured in organoids treated with cisplatin, LY411575 or amifostine, with the same tendency as the quantification of immunostaining images (Figure S5D). Then, we analyzed alteration in expression levels of progenitor cell marker and taste-associated genes in taste organoids after LY411575 treatment. Consistent with immunostaining data, expression levels of Lgr5 and Ki67, as well as Car4, PLCβ2, Snap25, NTPDase-2, T1R3 were enhanced by LY411575 in cisplatin-treated organoids (Figure 5E). Collectively, these data indicate that LY411575 alleviates the inhibitory effect of cisplatin on cell proliferation and taste receptor cell generation in organoids, similar to the cytoprotective agent amifostine.

### Pax1 and Pycr1 are potential targets to cisplatin and LY411575 treatment

**Figure 6:**
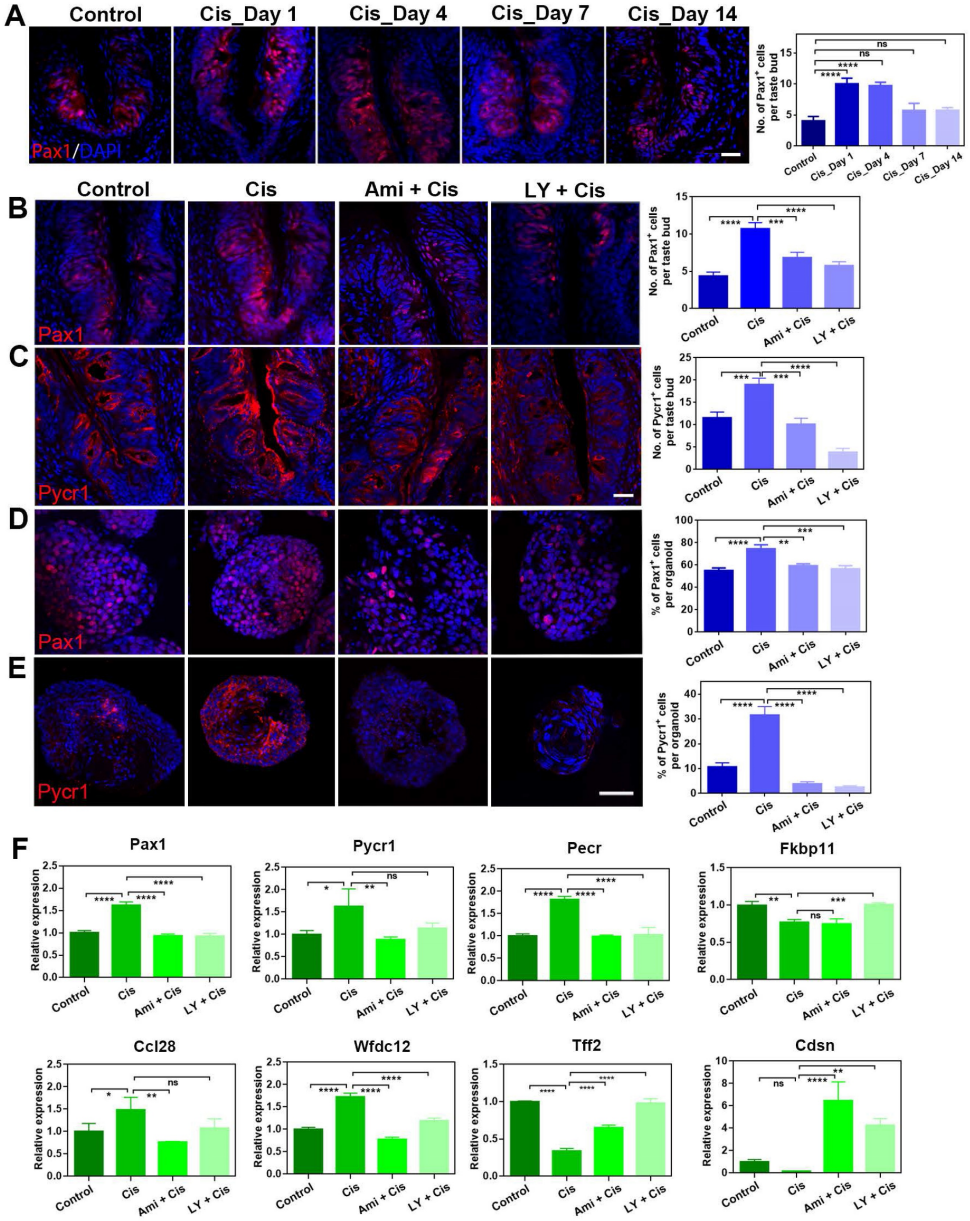
LY411575 counteracts the effect of cisplatin on Pax1 and Pycr1 expression in the circumvallate papilla and taste organoids. (A) Confocal images and quantification of the Pax1^+^ cells in the circumvallate papillae of saline and cisplatin-injected mice, at Day 1, 4, 7 and 14 post treatment. (B, C) Confocal images and quantification of Pax1^+^ (B), Pycr1^+^ (C) cells in the circumvallate papilla, injected with saline, cisplatin, cisplatin/amifostine, cisplatin/ LY411575. (D, E) Confocal images and quantification of Pax1^+^ (D), Pycr1^+^ (E) cells in the taste organoids, treated with saline, cisplatin, cisplatin/amifostine, cisplatin/LY411575. (F) Quantitative PCR showing expression levels of Pax1, Pycr1, Pecr, Fkbp11, Ccl28, Wfdc12, Cdsn, Tff2 in taste organoids treated with saline, cisplatin, cisplatin/amifostine, cisplatin/LY411575. ns, not significant, * p < 0.05, ** p < 0.01, *** p < 0.001, **** p < 0.0001, by one-way ANOVA with Dunnett's multiple-comparisons test. Scale bars, 25 µm in (A, C), 50 µm in (E).

To further validate the differential expression at protein level after cisplatin treatment, we immunostained circumvallate papilla sections with antibody against Pax1, an essential factor for normal T-cell maturation 34. The number of Pax1^+^ cells per taste bud was increased significantly by 152 ± 25% (p < 0.0001) and 147 ± 16% (p < 0.0001) at Day 1 and 4 post cisplatin treatment, compared to that in saline controls (Figure 6A), displaying consistent tendency with RNA-Seq and quantitative PCR data. Since cisplatin treatment regulated Pax1 expression in the circumvallate papilla, we then determined whether LY411575 counteracted cisplatin-induced change in Pax1 expression. The number of Pax1^+^ cells in the circumvallate papilla receiving cisplatin was decreased by 46 ± 5% (p < 0.0001) and 36±7% (p < 0.001) in the presence of LY411575 and amifostine, respectively (Figure 6B). Similarly, cisplatin increased the percentage of Pax1^+^ cells per organoid by 35 ± 7% (p < 0.001), while co-application of LY411575 or amifostine reduced the ratio of Pax1^+^ cells by 24 ± 4% (p < 0.01) or 20 ± 2% (p < 0.01) compared to that in cisplatin-treated organoids (Figure 6D). The effect of LY411575 against cisplatin-induced elevation in the number of Pax1^+^ cells was dose-dependent (Figure S5A, C), showing 23±14% (p > 0.05), 71±5% (p < 0.001), and 77±8% (p < 0.0001) decrease in the percentage of Pax1^+^ cells in organoids treated with 1 μM, 5 μM, and 10 μM LY411575, indicating specificity of the effect on Pax1. Thus, cisplatin elevates Pax1^+^ cell number in either taste buds or organoids, while LY411575 or amifostine counteracts this process.

Then, we determined whether Pycr1, a cooperator in cell apoptosis and protection, was regulated by LY411575. Pycr1 expression level in the circumvallate papilla was enhanced at either protein and mRNA levels after chemotherapy (Figure 6C, F). The number of Pycr1^+^ cells per taste bud was significantly raised by 64 ± 12% at Day 4 post cisplatin treatment (p < 0.01, Figure 6C). In the presence of LY411575 or amifostine, the number of Pycr1^+^ cells per taste bud was reduced by 80 ± 5% (p < 0.0001) or 47 ± 7% (p < 0.001) in the circumvallate papilla, compared to Pycr1^+^ cell number in cisplatin group (Figure 6C). Similar results were obtained in taste organoids, demonstrating an elevation by 201 ± 31% (p < 0.001) in the percentage of Pycr1^+^ cells per cisplatin-treated organoids than untreated ones, but reduction by 92 ± 5% (p < 0.0001) or 88 ± 7% (p < 0.0001) when co-applied with LY411575 or amifostine compared to cisplatin-treated organoids (Figure 6E). Furthermore, the expression changes of Pax1 and Pycr1 at protein levels was confirmed by western blot in taste organoids when treated with cisplatin, cisplatin/LY411575 or cisplatin/amifostine, showing the consistency between mRNA and protein levels (Figure S5D). Thus, LY411575 counteracts the effect of cisplatin on Pycr1 expression in either taste buds or organoids.

Finally, we analyzed alteration in expression levels of taste-associated and inflammation-associated genes in taste organoids after LY411575 treatment. LY411575 reduced the expression of inflammation-associated genes including Ccl28, Wfdc12, Pax1 in cisplatin-treated organoids (Figure 6F). LY411575, counteracting the effect of cisplatin, also increased expression levels of anti-apoptotic gene Fkbp11 35, epidermal barrier gene Cdsn 36, and tissue repair gene Tff2 37 in cisplatin-treated organoids (Figure 6F). Collectively, LY411575 regulates critical genes that are targets for cisplatin in taste buds and organoids.

## Discussion

In this study, we investigated the impact of cisplatin on taste cell proliferation, apoptosis and taste receptor cell generation, and identified potential targets and therapeutic reagents for taste disorders caused by cisplatin. Our findings have significant implications for establishing new strategies to restore taste function in patients receiving chemotherapy.

We hereby showed the role of alkylating agent cisplatin in the taste cell homeostasis and taste function. The effect of other alkylating agents on taste system has been reported previously. Cyclophosphamide enhanced TNFα expression in Type II but not Type III taste cells. In the same study, the authors also reported that amifostine reduced the amount of cyclophosphamide-induced TNF-α expression in taste buds 38. These data are consistent with our finding that cisplatin led to inflammatory activation, tested by inflammatory cytokine assay (Figure 3). However, we did not pinpoint which cell type(s) showed increase in inflammatory cytokine expression, while cisplatin impaired differentiation towards all three types of taste cells (Figure 2). Taste cells in different locations did not respond uniformly to cyclophosphamide since Types II and III cells in fungiform taste buds were more susceptible than circumvallate cells 39. Moreover, cyclophosphamide disturbed rapidly dividing cells in the basal layer of taste epithelium responsible for taste cell renewal. This validated our data that cisplatin treatment reduced the expression levels of progenitor cell markers Lgr5/Lgr6/Krt5 and cycling cell marker Top2a/Ki67 (Figure 2), suggesting that alkylating agents may target on cycling/progenitor/stem cells to impair the generation of taste cells.

The molecular mechanism underlying taste dysfunction caused by chemotherapy drugs remains poorly understood. Using RNA-Seq, we identified a group of differentially expressed genes (DEGs) between the circumvallate papilla of animals treated with saline and cisplatin. We found that the DEGs were significantly enriched in metabolic process, including the superoxide metabolic process. Noxa-1 acts upstream of or within the process of superoxide metabolic process and regulates redox signaling 40. Increased expression level of Noxa-1 after cisplatin treatment (Figure 4) may enhance the production of reactive oxygen species (ROS) and inflammation. This may explain the increasing number of apoptotic cells, restraints in cell proliferation and promoting inflammatory cytokine release in the circumvallate papilla after cisplatin injection. Cisplatin also decreased the expression level of insulin-like growth factor binding protein-3 (IGFBP-3) in taste buds (Figure 4). It has been reported that IGFBP-3 knockout led to increased TNF-α levels and apoptosis in retina 41, while increasing IGFBP-3 levels promoted recovery from retinal inflammation and apoptosis after ocular blast 42. Therefore, we hypothesize that the decreasing expression of IGFBP-3 after cisplatin treatment may aggravate apoptosis and inflammation in the taste bud.

Cisplatin treatment resulted in a temporary reduction in the expression of two genes associated with epidermal barrier. Corneodesmosin (Cdsn) is specific to desmosomes in epithelia undergoing cornification, and its knockout induced type 2 and type 17 inflammatory responses in skin 36. Cisplatin significantly reduced the expression level of Cdsn at Day 1 post-injection, suggesting possible early epidermal damage in the taste bud, which is recovered to some extent later. Stratifin (Sfn) is a cell cycle regulator responsible for epithelial keratinization and vital in maintaining epidermal homeostasis 43. Therefore, a decrease in Sfn expression level at Day 1 implies that cisplatin may transiently impair tissue homeostasis in the taste epithelium. The association between epidermal barrier breakage and taste cell differentiation will be further investigated.

Cisplatin-induced toxicity is closely related to inflammation, while reducing inflammation can alleviate tissue injury by cisplatin 44, 45. Our data revealed that cisplatin treatment led to differential expression of genes associated with inflammatory response in the circumvallate papilla. Lipopolysaccharide-induced inflammation upregulated Wfdc12 in epididymis 46. Accordingly, elevation of Wfdc12 expression level after cisplatin treatment in the circumvallate papilla is possibly resulted from enhanced inflammatory response. Ccl28 is involved in innate immunity and olfactory perception 47. The increased expression level of Ccl28 in the taste bud after cisplatin treatment suggests a boost in innate immunity. This is also supported by the previous finding of a potent antimicrobial activity of Ccl28 48. Furthermore, cisplatin also increased the expression level of Cxcl5, which accelerates inflammation 49 and recruits neutrophils 50. The elevated expression of Cxcl5 in cisplatin-treated circumvallate papilla may indicate neutrophil activation and enhanced inflammatory response. Therefore, the transcriptional alteration in the tongue induced by cisplatin treatment is associated with inflammatory activation and immune response.

Amifostine is a sulfhydryl drug that is used to protect healthy cells from damage caused by chemotherapy and radiation therapy. Studies using mouse models have demonstrated that amifostine has cytoprotective effects on taste buds. Pretreatment with amifostine prior to cyclophosphamide administration decreased the amount of TNFα expression in taste buds 38. Amifostine pretreatment also protected PLCβ2-and SNAP25-expressing cells in the circumvallate papillae from cyclophosphamide treatment 51. These data demonstrated that amifostine offset the detrimental effect of cyclophosphamide, via inhibiting inflammatory response and maintaining taste cell survival. LY411575 is a potent γ-secretase inhibitor and also inhibits Notch cleavage and has been shown to play a role in sensory cell differentiation 52. In taste organoid, LY411575 has been found to strengthen taste receptor cell generation and activate critical signaling pathways, including Wnt, PI3K-Akt, and MAPK 33. In cochlear organoids, LY411575 has been reported to facilitate hair cell generation, indicating a potential role in cell differentiation 53. To investigate whether LY411575 can protect against cisplatin-induced taste cell damage, we studied the effect of LY411575 as amifostine used in previous reports. Our findings demonstrated that LY411575 counteract the negative effects of chemotherapy, including apoptotic promotion and attenuation in cell proliferation and taste receptor cell generation in a dose-dependent manner (Figure 5, Figure S5). These results further support the role of LY411575 in taste cell proliferation and differentiation, and suggest that LY411575 may serve as a potential cytoprotective agent against chemotherapy-induced taste dysfunction. This effect of LY411575 is comparable to amifostine on alkylating agent-induced taste cell loss and inflammatory activation. Further investigation is required to understand the mechanisms underlying this cytoprotective effect of LY411575.

We proposed two genes, Pax1 and Pycr1, as potential targets to cisplatin and LY411575. Pax1 is necessary for function of the human thymus 54, and required for normal T-cell maturation 34, suggesting that cisplatin may affect T cell-regulated immune response, while LY411575 may adjust this process. Additionally, Pax1 plays a critical role in pattern formation during embryogenesis 55, implying that the pattern in taste bud may be altered after chemotherapy and restored by LY411575. Pycr1 responds to the cell protection against oxidative stress 56 and mediates proline metabolism to support cancer cell proliferation and survival 57. Cisplatin-induced upregulation of Pycr1 in either taste buds or organoids may reflect the self-protection of taste tissues to resist chemotherapy drug, while the excessive response is potentially restored by LY411575. To clarify the specific roles of Pycr1 and Pax1 in cisplatin/LY411575-induced taste cell alterations, experimental verification is necessary to provide direct evidences. Thus, this study identifies potential targets for chemotherapy drug-induced taste dysfunction, but further validation using animal and organoid models is essential before considering preclinical application. Our data suggest that LY411575 could be a potential treatment option for preventing the side effects of chemotherapy on the taste system, but additional research is needed to confirm its efficacy in clinical setting.

## Conclusion

In summary, we found cisplatin promotes apoptosis, inhibits cell proliferation and taste receptor cell differentiation in taste buds and organoids. The effect of cisplatin is potentially associated with regulation of inflammatory response, cell cycle and metabolic process. LY411575 counteracts the effect of cisplatin, while Pax1 and Pycr1 are regulated by LY411575/cisplatin in taste buds.

## Supplementary Material

Supplementary figures and tables.
