# Supplementary material for: Cisplatin attenuates taste cell homeostasis and induces inflammatory activation in the circumvallate papilla
Source: Theranostics. 2023 May 11;13(9):2896–913. doi: 10.7150/thno.81153 (PMC10240818; doi:10.7150/thno.81153)
Supplement: Supplementary file 1 — Supplementary figures and tables. [file thnov13p2896s1.pdf]

Supporting Information for

Cisplatin attenuates taste cell homeostasis and induces inflammatory activation in the circumvallate papilla

Wenwen Ren[1,#,*], Xudong Cha[1,#], Rui Xu[4,#], Tianyu Wang[1,#], Caiquan Liang[1], Janice Chou[5], Xiujuan Zhang[2], Fengzhen Li[1], Shenglei Wang[1], Boyu Cai[1], Peihua Jiang[5], Hong Wang[5,*], Huanhai Liu[1,*], Yiqun Yu[2,3,*]

[1] Department of Otolaryngology, the Second Affiliated Hospital of the Naval Military Medical University (Shanghai Changzheng Hospital), Shanghai, People's Republic of China.

[2] Ear, Nose & Throat Institute, Department of Otolaryngology, Eye, Ear, Nose & Throat Hospital, Fudan University, Shanghai 200031, People's Republic of China

[3] Olfactory Disorder Diagnosis and Treatment Center, Eye, Ear, Nose & Throat Hospital, Fudan University, Shanghai 200031, People's Republic of China

[4] School of Life Sciences, Shanghai University, Shanghai 200444, People's Republic of China

[5] Monell Chemical Senses Center, Philadelphia, PA 19104.

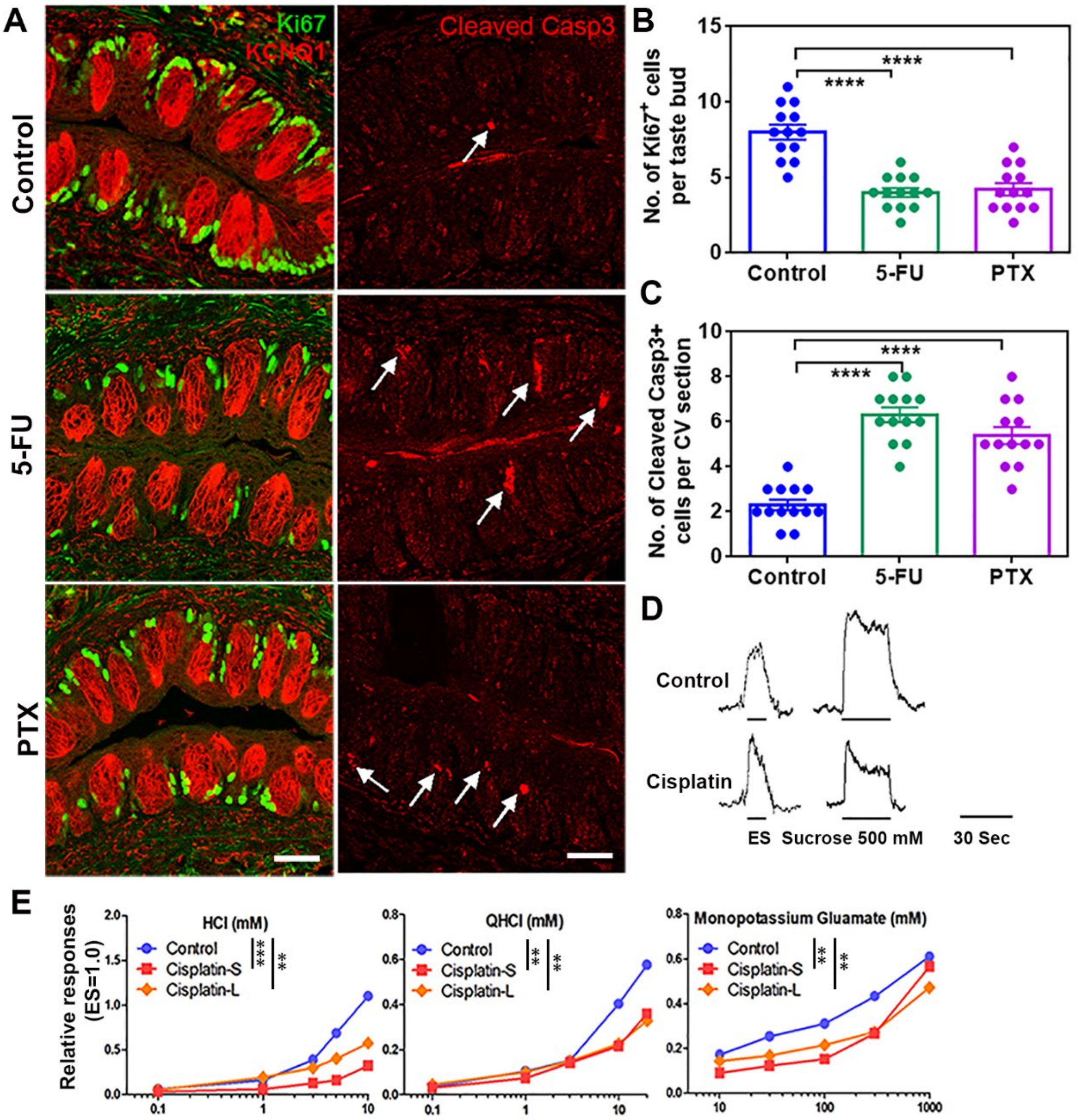

Figure S1. Chemotherapy drugs attenuate cell proliferation and promote apoptosis in the circumvallate papilla. (A) Immunostaining of Ki67 and KCNQ1, cleaved Casp-3 in the circumvallate papilla collected at Day 3 after injection of 5-FU or PTX. Arrows indicate positive cells. (B, C) Quantification of Ki67^{+}, cleaved Casp-3^{+} cells in the circumvallate papillae of control (PBS) and chemotherapy-drug-treated mice at Day 3 after injection. **** $p < 0.0001$ by one-way ANOVA with Dunnett's multiple-comparisons test. (D) Representative raw traces of whole-nerve recordings from chorda tympani nerves of mice with or without cisplatin treatment. ES, electric stimulation. (E)

Whole-nerve recordings from chorda tympani nerves of mice with or without cisplatin treatment. Mice received three doses of cisplatin or saline treatment via intraperitoneal injections either once every week (Cisplatin-S) or once every three weeks (Cisplatin-L). Data were integrated chorda tympani responses of control or treated wild-type mice in response to lingual application of a concentration series of HCl, QHCl, monopotassium glutamate, normalized to the response to electric stimulation (ES = 1.0). * $p < 0.05$, ** $p < 0.01$, *** $p < 0.001$, by two-way ANOVA with Sidak's comparisons test. Scale bars, 25 μm.

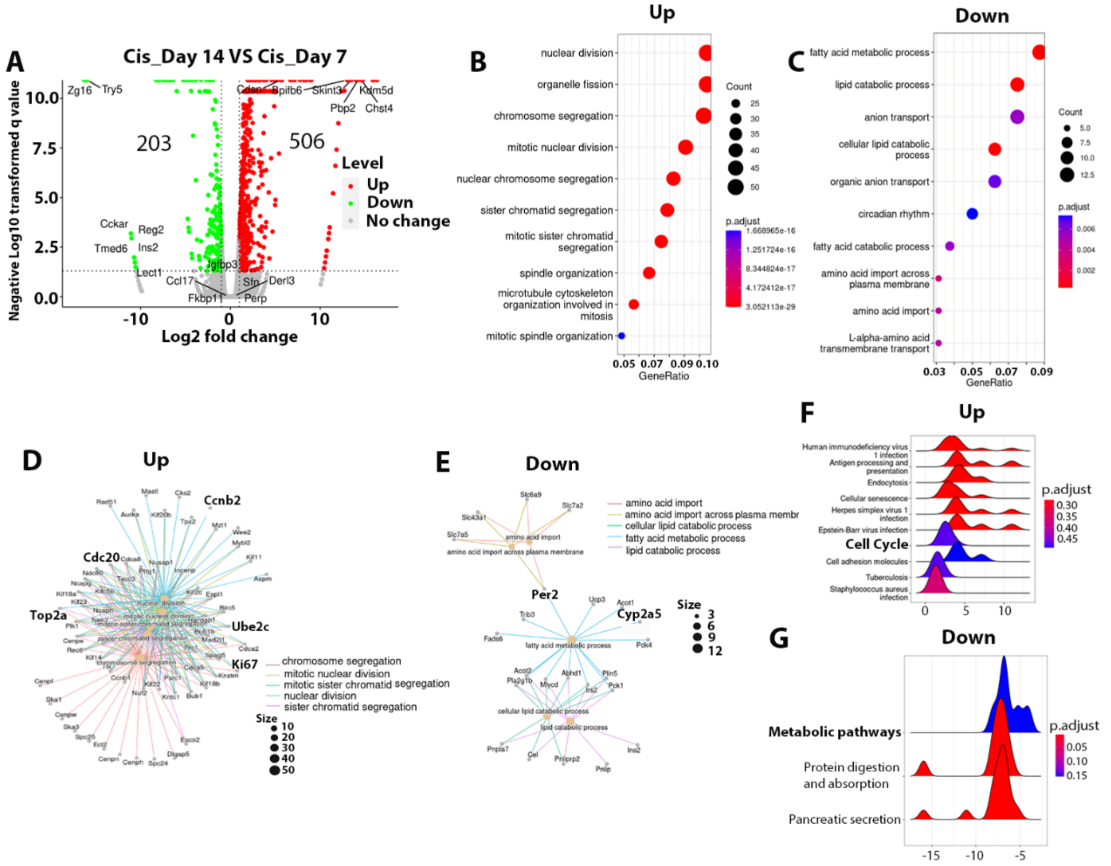

Figure S2. RNA-Seq analysis indicates the recovery of gene expression at Day 14 after cisplatin treatment. (A) Volcano plot showing the DEGs between the circumvallate papillae of cisplatin-injected mice at Day 14 and Day 7. (B, C) GO enrichment analysis of up- (B) and down-regulated (C) genes in the circumvallate papillae between Day 14 and Day 7 post cisplatin injection. (D, E) GO network analysis showing the critical genes involved in upregulated (D) and downregulated (E) GO terms. (F, G) Ridge plot of the GO terms of upregulated (F) and downregulated (G) genes in cisplatin-treated circumvallate papilla group at Day 14 compared to Day 7, defined by the GSEA.

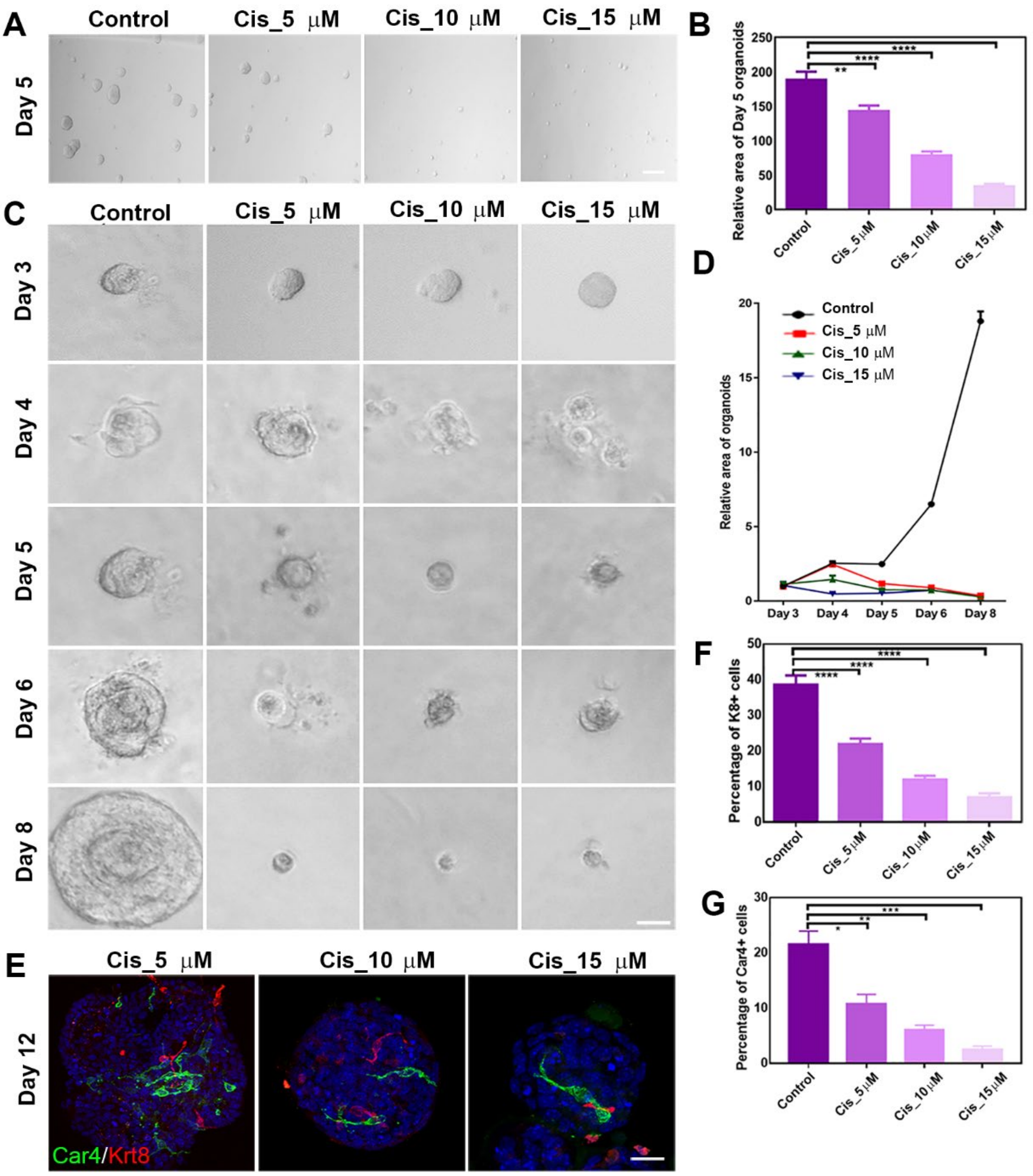

Figure S3. Cisplatin attenuates organoid growth and taste cell generation. (A) Microscopic images of taste organoids from circumvallate papilla at Day 5 post culture, with addition of cisplatin from Day 0. (B) Quantification of organoid size at Day 5, with 0, 5, 10, or 15 μM cisplatin added at Day 0. ** $p < 0.01$, **** $p < 0.0001$, by one-way ANOVA with Dunnett's multiple-comparisons test. (C) Microscopic images of taste organoids from Day 3 to Day 8 post culture, with cisplatin added at Day 3 and incubated for 48 h. (D) Quantitative analysis of organoid size from Day 3 to Day 8, with cisplatin treatment from Day 3 to Day 5. (E) Confocal images of $Krt8^{+}$ and $Car4^{+}$

cells in taste organoids at Day 12 post culture, with cisplatin added at Day 4 and incubated for 8 days. (F, G) Quantification of Krt8$^+$ (F) or Car4$^+$ (G) cells in taste organoids at Day 12, with cisplatin added at Day 4 and withdrawn at Day 12. * $p < 0.05$, ** $p < 0.01$, *** $p < 0.001$, **** $p < 0.0001$, by one-way ANOVA with Dunnett's multiple-comparisons test. Scale bars, 50 μm.

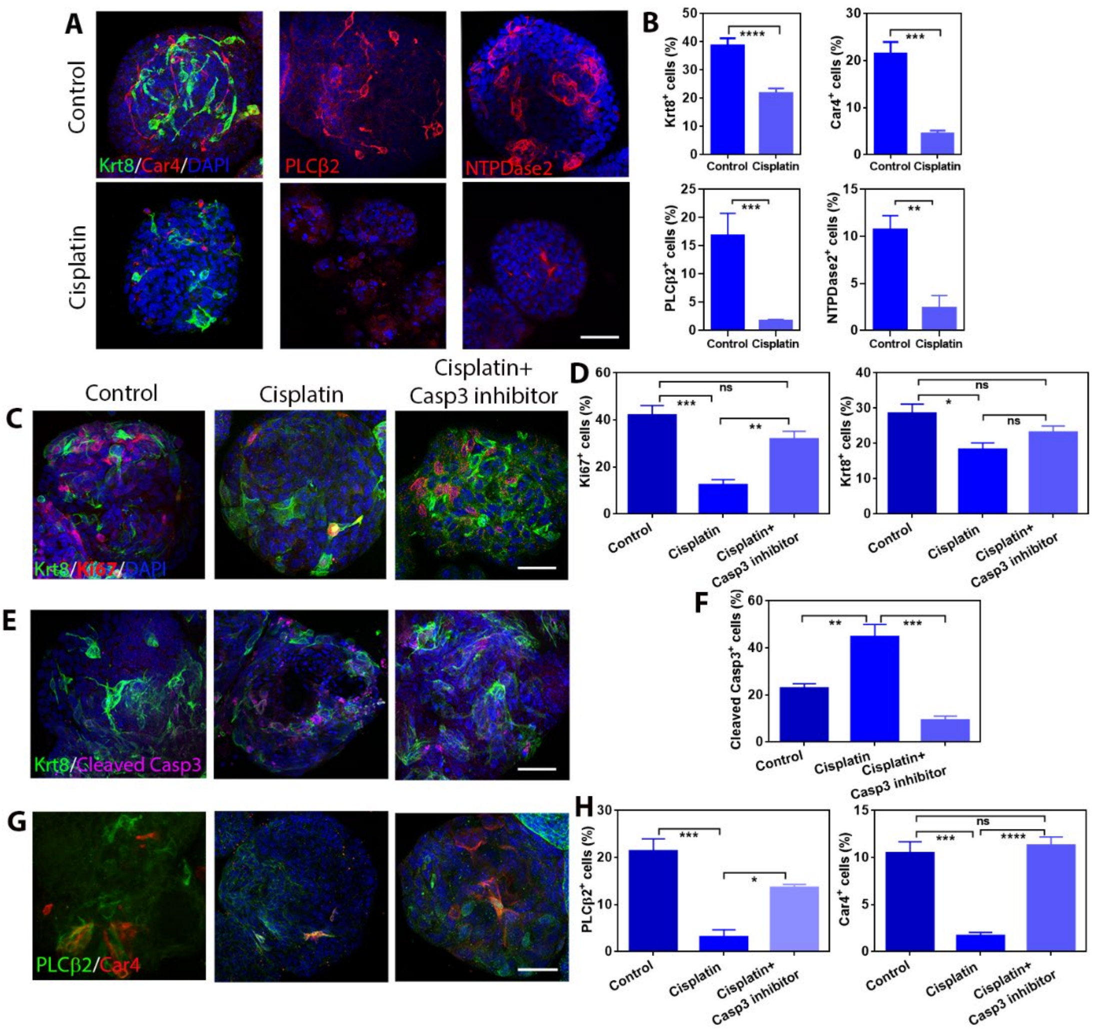

Figure S4. Caspase 3 inhibitor promotes cell proliferation and taste cell generation in cisplatin-treated organoids. (A) Confocal images of Krt8^{+}, Car4^{+}, PLCβ2^{+}, NTPDase2^{+} cells in taste organoids treated with saline or 5 μM cisplatin. (B) Quantification of Krt8^{+}, Car4^{+}, PLCβ2^{+}, NTPDase2^{+} cells in taste organoids treated with saline or cisplatin. ** $p < 0.01$, *** $p < 0.001$, **** $p < 0.0001$, by unpaired t test. (C, E, G) Confocal images of Krt8^{+}/Ki67^{+} (C), cleaved Caspase 3^{+} (F), PLCβ2^{+} and Car4^{+} (G) cells in organoids treated with saline, cisplatin, cisplatin/Caspase 3 inhibitor. (D, F, H) Quantification of Krt8^{+}, Ki67^{+} (D), cleaved Caspase3^{+} (F), PLCβ2^{+}, Car4^{+} (H) cells in organoids treated with saline, cisplatin, cisplatin/Caspase3 inhibitor. ns, not significant, * $p < 0.05$, ** $p < 0.01$, *** $p < 0.001$, **** $p < 0.0001$, by one-way ANOVA with Dunnett's multiple-

comparisons test. Scale bars, 50 μm.

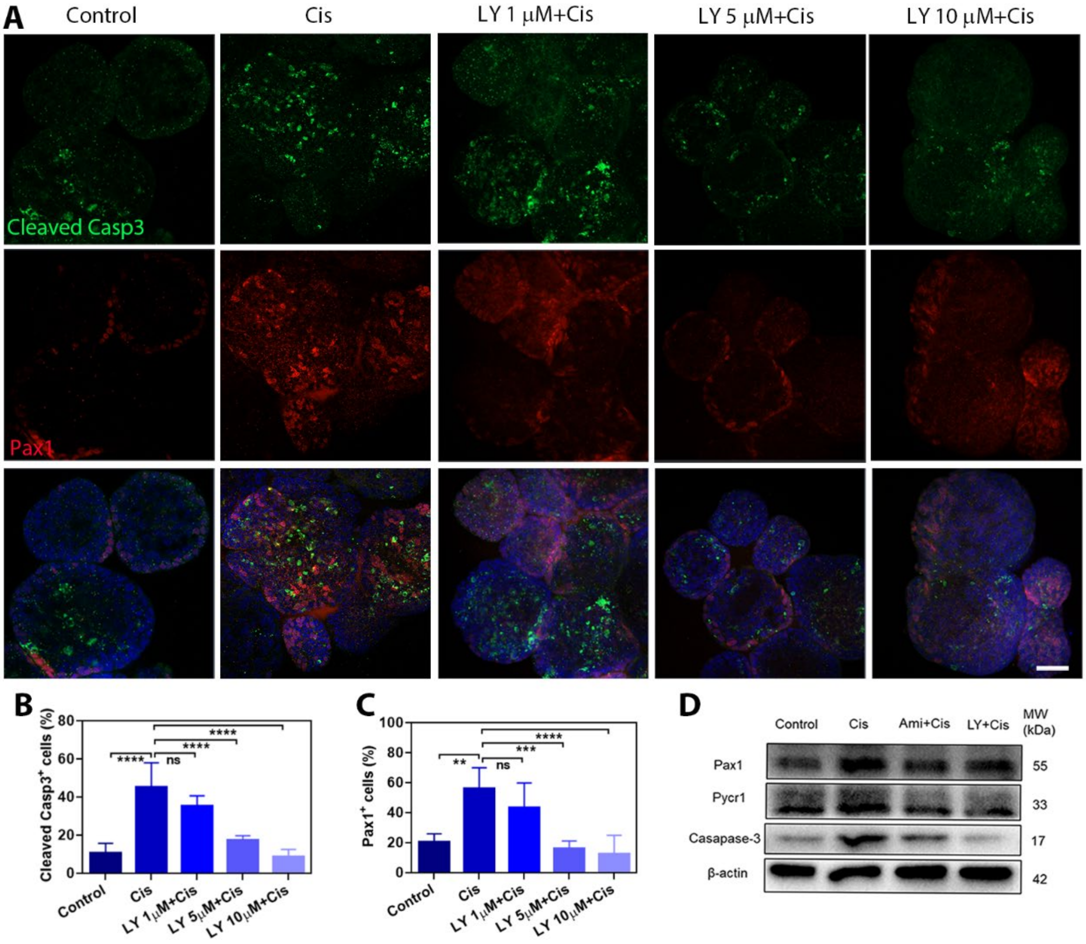

Figure S5. The counteractive effect of LY411575 in cisplatin-treated taste organoids is dose-dependent. (A) Confocal images of cleaved Caspase 3^{+} cells, $Pax1^{+}$ cells in taste organoids treated with saline, cisplatin, cisplatin and LY411575 at 1, 5, 10 μM. (B, C) Quantification of cleaved Caspase 3^{+} (B), $Pax1^{+}$ (C) cells in organoids treated with saline, cisplatin, cisplatin and LY411575 at 1, 5, 10 μM. ns, not significant, ** $p < 0.01$, *** $p < 0.001$, ****$p < 0.0001$, by one-way ANOVA with Dunnett's multiple-comparisons test. (D) Western blot showing the changes in expression levels of cleaved Caspase 3, Pax1, Pycr1 in taste organoids treated saline, cisplatin, cisplatin/amifostine, cisplatin/LY411575. Scale bar, 50 μm.

Table S1. Antibodies used in the current study.

Antibody	Host	Cat.No.	Dilution (x)	Source
KCNQ1	Goat	sc-10646	1000	Santa Cruz
Cleaved Caspase 6	Rabbit	9761S	100	CST
Cleaved Caspase 3	Rabbit	9661	100	CST
Ki67	Rabbit	ab16667	300	Abcam
Gustducin	Rabbit	sc518163	500	Santa Cruz
Car4	Goat	AF2414	20	Abcam
Pax1	Rat	ab252847	500	Abcam
Pycr1	Rat	AB_102601	500	Abcam
Krt8	Rat	AB_531826	10	Developmental Studies Hybridoma Bank
NTPDase2	Rabbit	AB_2314986	500	Centre de Recherche du CHUL
Plcb2	Rabbit	SC_515912	100	Santa Cruz
Anti-rabbit IgG-Alexa 488	Donkey	A21206	500	ThermoFisher
Anti-rabbit IgG-Alexa 647	Donkey	A31573	500	ThermoFisher
Anti-goat IgG-Alexa 594	Donkey	A11058	500	ThermoFisher
Anti-goat IgG-Alexa 633	Donkey	A21082	500	ThermoFisher
Anti-rat IgG-Alexa 647	Donkey	AB150155	500	Abcam

Table S2. Primer sequences for quantitative PCR.

Gene	Forward primer	Reverse primer
Wfdc12	TGACGCTCCTTATTTCCTCTACT	GTAACACTGGGGATCATCTTGC
Bpifa2	TGGGAACTCAGAGTCACTTCTT	TGCTGAAGCAACTCCACATCC
Adora1	TGTGCCCGGAAATGTACTGG	TCTGTGGCCCAATGTTGATAAG
Pax1	CCGCCTACGAATCGTGGAG	CCCGCAGTTGCCTACTGATG
Rab26	TTGGTAGCACTGGAGACTTCT	CCATCCTTGAAGCGCACAA
Fkbp11	CGAAGAGCAGTCATTCCTTCTC	TGGCTCGAATCAAAGCGATCA
Pycr1	ATGAGCGTAGGCTTCATCGG	GTGTCAGGTTCACCCCTATCT
Ralyl	AAAGCCTGGAAGTAAGAGACCC	GGAATGGCTCCTTTGATTCAAGC
Pecr	GGTCAGAGCTACCTAGCGG	GACCACGTTACACCCCAGG
Igfbp3	CACACCGAGTGACCGATTCC	GTGTCTGTGCTTTGAGACTCAT
Perp	ATCGCCTTCGACATCATCGC	CCCCATGCGTACTCCATGAG
Sfn	CCTGCTTTCCGTAGCTTACAA	CTCGTTGCTCTTCTGCTCGAT
Derl3	ATGCTGGTCTATGTATGGAGCC	GTAAGCCGAAGAAGTTGACCC
Lgr5	TAAAGACGACGGCAACAGTG	GATTCGGATCAGCCAGCTAC
Car4	TGGCTCACTAACCACACCAA	GGCCTCACATTGTCCTTCAT
GAPDH	TCAATGAAGGGGTCGTTGAT	CGTCCCGTAGACAAAATGGT
